# Food Consumption Determinants and Barriers for Healthy Eating at the Workplace—A University Setting †

**DOI:** 10.3390/foods10040695

**Published:** 2021-03-25

**Authors:** João P. M. Lima, Sofia A. Costa, Teresa R. S. Brandão, Ada Rocha

**Affiliations:** 1 Politécnico de Coimbra, ESTeSC, Unidade Científico-Pedagógica de Dietética e Nutrição, Rua 5 de Outubro, S. Martinho do Bispo, 3046-854 Coimbra, Portugal; 2 GreenUPorto—Sustainable Agrifood Production Research Centre, Campus de Vairão Edifício de Ciências Agrárias (FCV2) Rua da Agrária, 747, 4485-646 Vairão, Portugal; adarocha@fcna.up.pt; 3 LAQV-Requimte—R. D. Manuel II, Apartado 55142, 4051-401 Porto, Portugal; 4 ciTechCare—Center for Innovative Care and Health Technology, R. de Santo André 2410, 2410-541 Leiria, Portugal; 5 Instituto de Saúde de Pública da Universidade do Porto, Rua das Taipas 135, 4050-091 Porto, Portugal; sofcosta1@sapo.pt; 6 CBQF—Center for Biotechnology and Fine Chemicals—Associate Laboratory, School of Biotechnology, Catholic University of Portugal, R. de Diogo Botelho 1327, 4169-005 Porto, Portugal; tbrandao@porto.ucp.pt; 7 Faculty of Nutrition and Food Sciences, University of Porto, Rua do Campo Alegre, 823, 4150-180 Porto, Portugal

**Keywords:** food choice, food consumption, university, workplace, determinants, barriers

## Abstract

Background: A wide variety of social, cultural and economic factors may influence dietary patterns. This work aims to identify the main determinants of food consumption and barriers for healthy eating at the workplace, in a university setting. Methods: A cross-sectional observational study was conducted with 533 participants. Data were obtained through the application of a self-administered questionnaire that included socio-demographic information, food consumption determinants and the main perceived barriers for healthy eating at the workplace. Results: The respondents identified “price” (22.5%), “meal quality” (20.7%), and “location/distance” (16.5%). For women, the determinant “availability of healthy food options” was more important than for men (*p* < 0.001). The food consumption determinants at the workplace most referred to by respondents were related to the nutritional value. Smell, taste, appearance and texture, and good value for money, were also considered important for choosing food at the workplace. Respondents referred to work commitments and lack of time as the main barriers for healthy eating at the workplace. Conclusions: Identification of determinants involved in food consumption, and the barriers for healthy eating, may contribute to a better definition of health promotion initiatives at the workplace aiming to improve nutritional intake.

## 1. Introduction

Globalization has caused drastic changes in food patterns within the last decade. These changes resulted in a reduction in the prevalence of malnutrition along with a widespread increase in prevalence of overweight and obesity [1]. An unhealthy lifestyle is one of the major risk factors for chronic diseases in developed countries [2]. Consumer behaviors play a prominent role in the etiology of several chronic non-communicable diseases, including obesity, diabetes mellitus, and cardiovascular diseases, among others, whose prevalence tends to stand still, or even increase [1,3,4].

Sedentary habits and unhealthy eating behaviors are responsible for a significant economic burden through absenteeism and presenteeism [5,6,7,8]. Additionally, for employees, unhealthy lifestyle behaviors and obesity might lead to negative effects related to work [9]. Research has shown that unhealthy employees and those with an unhealthy lifestyle are less productive at work and have decreased work ability [10,11,12,13,14].

The workplace is recognized as an opportune and fruitful setting for health promotion because of the presence of natural social networks, the possibility of reaching a large number of people, and the amount of time people spend at work [15,16]. Promotion of healthy lifestyles, namely healthy nutritional behavior at the workplace, improves workers’ health and productivity [17].

The workplace also offers an interesting context for studying eating behaviors. There is often a high level of consistency in people’s working lives, with many workers (particularly those who are office-based, as in this sample) spending most of their time in the same location surrounded by the same group of colleagues [18]. Partly for this reason, a number of eating-related research studies have been conducted at the workplace [19,20,21].

A wide variety of social, cultural, and economic factors may influence dietary patterns. Intra-individual determinants, such as physiological and psychological factors, acquired food preferences, and knowledge about nutrition can be distinguished from interpersonal or social factors, such as family and partners influence [21].

Food choice determinants are frequently presented in four groups:

(a) Biologically determined behavioral predispositions, related to an individual’s innate abilities related to food, namely the preference for sweet and salty foods; the mechanisms that control hunger and satiety; and the sensory experience provided by food. These are the most basic determinants of food choice, meaning when choosing food or drinks, people firstly follow their preferences [21];
(b) Sensory-affective factors—those related to feelings and emotions in relation to food—acquired familiarity and ability to learn how to like something are at the second level [21];
(c) Intrapersonal factors, defined by an individual’s beliefs, attitudes, knowledge, skills and social norms, follow the previous factors in determining the choice of food, just like the interpersonal ones, which involve family, friends and other social networks [21]. The culture in which each individual was born and raised influences general behavior and food habits [21]. Interpersonal factors theoretical framework was also described by Rothschild, 1999 [22], and applied, for example, in Bos, 2016 [23]. Several authors have ascertained that choices depend on the surrounding environment, and are based on one’s knowledge and experience [21];
(d) Environmental factors are the last level determining food consumption. Even though they are the most distant from the individual, environmental factors are the easiest to influence. They include availability and accessibility to food; social, environmental and cultural practices; resources; economic environment; and food marketing practices [21]. For example, resources and economic environment determine food consumption through food cost or individual income [21]. According to the literature, low-income population groups are more likely to adopt unbalanced diets [21].

In addition to the determinants described above, the individual’s psychological state is also assumed as one of the major determinants of the act of eating. Situations of emotional difficulty, states of anxiety and stress, situations of rejection, or loneliness, in more vulnerable individuals, can lead to changes in eating behavior [21].

Several studies concluded that individuals who identified a higher number of barriers for healthier eating habits correspond to those with worse habits [23,24]. The main factors identified by consumers as barriers for healthy eating were lack of time, poor cooking skills, food price, or the lack of healthy choices at food service units [23,24,25,26].

Meals eaten at the workplace represent a large contribution to the daily energy intake and influence the balance of the diet [27]. The study “Food and Portuguese Population Lifestyle” [28], identified the factors that influence the food choices of Portuguese adults, and their relationship with socio-demographic and health features [29]. The attribute of “Taste” was the most important factor determining food choice, followed by the “Price” and the “Intention of healthy eating”, according to Poínhos et al. [29].

Previous research conducted at different workplaces related to food consumption determinants and perceived barriers, identified that structures and systems within the workplace have a significant role in dietary behaviors. These include the facilities available [30,31,32], training of staff [33], long hours worked as a result of high workloads and work pressures, and a culture that encourages working through breaks [34,35]. Lack of time for lunch can affect both health and productivity [36,37]. The conflict between promoting a greater range of healthier foods and business constraints has also been previously identified [38].

In order to develop effective workplace interventions for healthy eating, researchers must first consider all the known determinants of eating behavior as potential targets for intervention, such as distinct features of working conditions. In a recent systematic review of factors affecting healthy eating among nurses, the majority of studies found that workplaces often create barriers for healthy eating [20]. Therefore, to define appropriate health promotion initiatives, it is necessary to characterize the determinants involved in food choice, in order to influence food consumption at the workplace. Additionally, to identify perceived barriers for healthier eating habits it is also important for the implementation and assessment of interventions in different scenarios [39,40].

To the best of our knowledge, there are no studies that identify and characterize the determinants involved in food choice in Portugal, especially at the workplace, and it becomes relevant to develop research to better understand this subject. Therefore, this study intends to identify the perceived barriers for healthy eating, and the main determinants of food consumption at the workplace, among university employees.

## 2. Materials and Methods

### 2.1. Study Design and Sample

A cross-sectional observational study was conducted at a Portuguese university through face-to-face interviews by a trained researcher at the participants’ workplace. This university had 3307 employees: 1750 teachers and researchers (academic), 1551 non-teaching staff (non-academic) [41]. A convenience sample was used, stratified by organic units, aiming to represent the study population, allowing researchers to infer conclusions for the study population. Given that the sample corresponds to approximately 15% of the population, it was stratified into teaching and researcher staff, and non-teaching and non-researcher staff; 533 employees were selected. Data collection was performed during labor hours.

### 2.2. Ethical Issues

The project was approved by Ethical Commission of the University of Porto, with the number CEFADE 25.2014. The principles of the Helsinki Declaration were respected and the workers under analysis accepted participation in the study through informed consent, after having the purpose and methods involved in the study explained to them individually.

### 2.3. Questionnaires for Data Collection

Data were obtained through the application of a self-administered questionnaire. It included socio-demographic information and food consumption determinants at the workplace, and a list of barriers for healthy eating at the workplace. The questionnaire included questions such as the employee’s age, gender and marital status. Academic qualifications were also questioned, through a closed answer format composed of nine levels of response (between primary school and PhD or Post-Doc). Employees with academic qualifications higher than bachelor’s degree were asked about the training area. Concerning work practices, respondents were asked about the amount of time they spend working at this institution, and the work regime (full-time or part-time). They were asked about the professional category, function performed, with discrimination between teaching and non-teaching activity, and the establishment where they work.

To assess food consumption determinants, a section of the questionnaire was developed through the adaptation of the Food Choice Questionnaire, developed by Steptoe et al. [42] after translation and validation for the Portuguese population by Cardoso and Vale [43]. Steptoe et al. also contributed to the questions of the Food Choice Questionnaire. A Likert Scale of 5 points, from strongly disagree (1) to strongly agree (5) was used in the questions related to determinants. Questions used in the studies “Food and Portuguese Population Lifestyle” and “Food and Portuguese Population Lifestyle” [28,29] were included in the questionnaire. The determinants of the choice of location for lunch in the workplace were also evaluated. Respondents were invited to select the three main factors affecting their choice from a predefined list presented in our results [29,44,45,46,47].

The barriers presented to respondents were selected from the literature, and others were added considering individual perceptions of the researchers. Respondents could select as many options from the list as they wanted.

Food offer, quality of meals, prices and food and nutritional intake of employees were analyzed and published in previous research papers [48,49].

### 2.4. Statistical Analysis

Data were analyzed using the Statistical Package for Social Sciences version 21.0 ^®^ for Windows. Descriptive analysis was performed, and normality of cardinal variables was tested with Shapiro-Wilk Test. Association between nominal variables was analyzed by chi-square test. Association between ordinals and nominal variables was performed with Kruskal-Wallis tests. Between ordinal variables, or between ordinal and cardinal non-normal, Spearman correlation was performed. Taking into consideration the differentiation of the sample in terms of age, results were analyzed by age groups, through splitting the sample by the median age (43 years old) to identify younger and older respondents. Cut-off of 0.05 was used as the level of statistical significance. Data were also analyzed according to Multiple Correspondence Analysis (MCA) procedures, which allows for exploring the pattern of relationships of several categorical variables and representing them in few dimensions of homogeneous variables. For this model, sociodemographic variables were included, namely gender, educational level, and professional occupation; lunch setting (lunch brought from home, university food services, restaurants and go home), determinants for the lunch place choice and determinants of food consumption identified from Food Choice Questionnaire [42,43].

## 3. Results

### 3.1. Sample Characterization

From 533 assessed individuals, 513 were considered valid answers. Participants were aged between 21 and 80 years old (mean 43.3 ± 10.6), mostly females (65.5%) and married (63.4%). About 94% of respondents were full-time workers. Most workers (80.3%) had a university degree and about 35% had a PhD or a Post-Doc diploma. Only 3.3% of respondents did not complete high school education. Of respondents, 34.2% were Teachers, 63.0% were Non-Academic Staff/Researchers and 2.8% had both activities.

The majority of workers had a sedentary activity since 81.5% of them reported spending most of their time seated, and 74.5% characterized their work as not being “very physically demanding”.

Only 23.1% of respondents reported following an unhealthy diet at the workplace. Hence, only these workers were asked to point out the barriers for adopting a healthier diet.

### 3.2. Determinants of Choosing the Place for Having Lunch

The majority (96.7%) of respondents had lunch every day, however, only 36.1% of them attended the university food service. Of the respondents, 28% had lunch in local restaurants. About 52% of workers brought lunch from home and only 16.2% had lunch at home.

**Figure 1:**
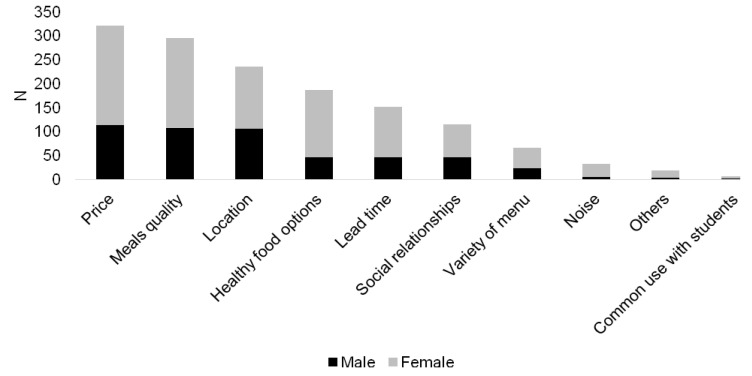
Food consumption determinants to choose the place for having lunch per gender. N: Number of individuals

The respondents identified “price” (22.5%), “meal quality” (20.7), “location/distance” (16.5%), “healthy food options” (13.1%) and “lead time” (10.6%) as the most important determinants used to choose the place for having lunch. For women, the option of having “healthy food options” (*p* < 0.001) was more important than for men. Additionally, “location” (*p* < 0.001) and “noise” (*p* = 0.016) were more important for women than for men (Figure 1).

“Price” as a determinant for choosing the place for having lunch was more important in younger respondents (Table 1). This determinant was also more important for those with a lower academic degree (*p* < 0.001) than for those with a higher level of education. Respondents with a higher academic degree referred more frequently to “Location/Distance” of places for having lunch as a determinant of choice. “Meal quality” (*p* = 0.002) and “healthy food options” (*p* = 0.049) were considered determinants for choosing the lunch setting more frequently by teaching staff.

**Figure 2:**
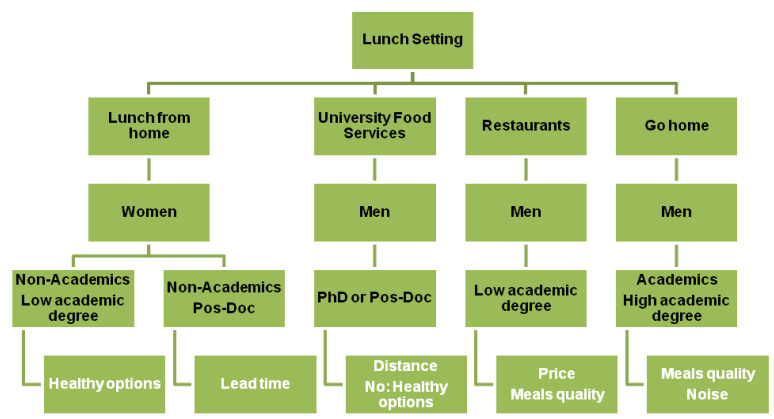
Food consumption determinants to choose the setting for having lunch (Multiple Correspondence Analysis (MCA) analysis).

Based on results of MCA three main dimensions were identified that explained 33.4% of data variability. The following homogeneous groups of variables were obtained (Figure 2).

### 3.3. Determinants of Food Consumption at the Workplace

Determinants of food consumption at the workplace most referred to by respondents (more than 70%) were related to foods rich in vitamins, minerals and fiber, nutritionally balanced, with natural ingredients and no additives, and that contribute to health and weight control. Smell, taste, appearance, texture, and a good value for money were also considered important for choosing food at the workplace.

**Figure 3:**
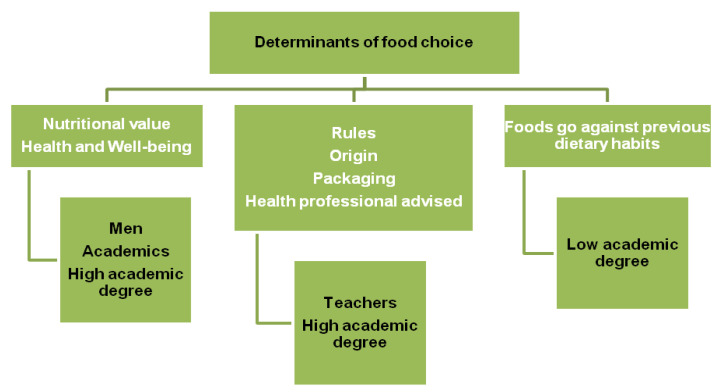
Food consumption determinants at the workplace (MCA analysis).

Based on the results of MCA, two main dimensions were identified that explained 59.9% of data variability, and the following homogeneous groups of variables were obtained (Figure 3).

### 3.4. Barriers for Healthy Eating at the Workplace

**Figure 4:**
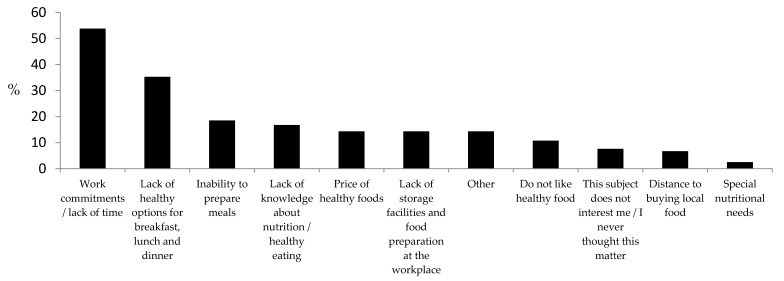
Frequency of perceived barriers for healthy eating at the workplace.

The participants referred mostly to work commitments and lack of time as barriers for healthy eating at the workplace (Figure 4). From the barriers under analysis, differences between genders were only observed related to knowledge about nutrition. Males identified “Lack of knowledge about nutrition/healthy eating” as a barrier for healthy eating more frequently than women (Table 2). No differences were observed between age groups related to perceived barriers for healthy eating (Table 3).

In comparing academic with non-academic respondents, significant differences for two distinct barriers were found. It seems that food price is a prohibitive factor for having a healthy diet, essentially for non-academic staff in relation to other individuals (*p* = 0.004). Lack of healthy options for breakfast, lunch and dinner were identified by academic staff more frequently than by non-academics (*p* = 0.012) (Table 4). Concerning other parameters assessed, ranges of age and marital status did not seem to influence the barriers for healthier eating at the workplace.

## 4. Discussion

Major determinants for choosing a place to have lunch were related to “meal quality”, “price”, and “location”. Working at higher education institutes determines an increased burden of work and responsibilities, most of them extra classes [50], which contributes to work commitments and lack of time to take breaks, prepare, and have healthy meals. Additionally, sensory aspects of food consumption can influence the choice of lunch place. Sensory aspects are usually observed as determinant of food consumption. The cost of meals is more relevant for younger respondents as observed in a previous study [51].

Younger, non-teaching female employees with lower academic qualifications are the group who most frequently bring lunch from home. Bringing food from home is likely associated with higher level cooking skills—more common in the female gender [25]. Additionally, this group also has lower disposable income and hence, bringing food from home allows for more savings.

Lunch location is also determined by other factors. According to other authors, meals outside the home often have a higher energy value and a poorer nutritional profile [27]. Indeed, of the women who bring lunch from home, some do so to ensure a healthier lunch.

On the other hand, teachers with PhD or Post-Doc Diplomas mentioned waiting time as a key decision driver. This is likely associated with a higher level of responsibility, strong focus on work, and consequently, shorter lunch breaks.

In this study, food availability was identified more frequently by academic staff than other respondents. On the other hand, non-academics reported a higher concern, and identified the lack of storage facilities and food preparation areas at the workplace as a barrier. This parallelism on identified barriers could indicate that academics more frequently use university cafeterias, and non-academics bring food from home and use storage and preparation facilities, when available at the workplace, more frequently. These results are in line with the identification of a third barrier, significantly the difference between individuals with different professional occupations. Effectively, non-academics identified the price of healthy food options as a barrier for healthy eating more frequently than academics. Differences in salary between them could explain this result. The perception of these factors could influence the choice of place for having meals—cafeterias, or storage and preparation facilities.

Attending to the wide availability of information about healthy eating, the number of respondents that identify the lack of knowledge about nutrition or healthy eating as a barrier is unexpected. Men identified this barrier more frequently than women. In addition, Yahia observed that men identified the barrier, lack of knowledge about nutrition or healthy eating, more frequently than women, among university students [52].

Universities are a captive environment where staff is restricted to a campus where offices, classes and study facilities are located, and where there is limited choice for food provision [53,54]. The workplace can be a strong determinant of food consumption behavior as it provides convenient access to healthy and/or unhealthy food choices. In a population experiencing time constraints having good food choices at the workplace provides an easy option for refueling [37,48]. Food available at, or near workplaces, is more convenient, low in cost, and sells well [21]. Similar findings were reported by Pinhão et al. in a representative sample of the Portuguese population, where “taste” was the most selected factor, followed by “price” and “trying to eat healthy” [29] as determinants of food choice.

Our results are in accordance with those found by Kjøllesdal in Norwegian adults, showing that people with higher educational levels and in higher income groups ate in staff canteens more frequently than others [55].

According to previous literature, access to healthy foods in the workplace is often limited, compared with an abundance of unhealthy foods present in workplace canteens, onsite shops, and vending machines [46,48,56,57]. According to literature, workers desire a greater variety of healthy and fresh foods compared with the current offerings [46,57,58,59], which is identified in this research as a barrier for healthy eating. Healthy options also determined workers food choice. Interestingly, some employees felt that food served in the canteen is not balanced with their nutritional needs. The factors that influence food consumption of employees related to healthy options, nutritional value of foods, meal quality, and health and well-being, may be associated with employees’ perception of canteen’ meals being too high in calories and tailored for physically demanding roles [46].

However, employees also reported that the lunch provided by the work canteen is the only opportunity to have a “proper meal” each day [58]. In the same way, the workplace could be a provider of healthy foods (such as vegetables and fruit) and increase intake of those foods [59,60]. Availability at the workplace is a determinant for food choice and a barrier for healthy eating, the reasons why the availability of facilities where food can be prepared was considered to be an important facilitator of healthy eating [46,59]. On the other hand, the higher cost of healthy options compared with unhealthy options was identified as one of the most significant barriers to healthy eating [46].

The determinants that most influence food choice at the workplace in this study are related to the individual. The identification of knowledge about the health benefits of food is commonly observed, followed by biological determinants such as taste, smell, or the texture of the food, and finally, of an environmental nature related to the quality-price ratio of the food.

Food choices of men, with higher academic qualifications and belonging to the teaching staff, are determined by food taste and texture, and by availability and price-quality relationship. Additionally, they value the potential benefits of food, and their food choice is determined by them. The influence that foods can have on well-being is also important, such as choosing foods that help maintain alertness and support emotional health.

Regardless of gender, among professors with higher academic qualifications, food choice is determined by cultural, religious or ethnic beliefs, political ideologies, the clarity and environmental responsibility of packaging products, and medical advice regarding the intake of certain foods. On the other hand, among individuals with lower academic qualifications, these determinants have a reduced importance.

In fact, food choice is a complex result of preferences for sensory characteristics, combined with the influence of non-sensory factors, including food-related expectations and attitudes, health claims, price, ethical concerns and mood, as already reported by other authors [47,61]. Regarding these concerns, the availability of healthy food options at the workplace, namely in cafeterias, is very important. On the other hand, the inability to prepare meals was also identified as a barrier for healthy eating, pointing to a need to improve cooking skills, for example, by the inclusion of this topic in the school curriculum.

Only a small proportion of respondents perceived barriers for adoption of a healthy diet. Other authors observed similar results [25,26,62]. Healthier environments should be promoted to facilitate healthy eating and fighting chronic diseases such as obesity [63]. However, of all variables tested, only the price and lack of knowledge about nutrition/healthy eating showed significant differences between respondents. Some studies have shown that people that identify a higher number of barriers are those that follow unhealthy eating habits more frequently [24,63].

The barriers identified in this research are related only to individuals that are considered as having unhealthy eating at the workplace. Future works should also include those who are considered as having healthy habits.

Strategies to promote healthier food habits aim at reducing barriers to access healthy options and increasing opportunities for employees to make healthier food choices. Implementation includes provision of healthier options, improved accessibility, and establishment of mandatory policies to provide healthy options or restrict less healthy offerings at the workplace [16].

Some limitations were identified in this study. Lack of information concerning income that impair conclusions potentially explained by this. Another limitation was related to the usage of different tools to access food determinants for choosing the place to have lunch, and the determinants of food consumption in general. However, the fact that the tool used to access the determinants for choosing the place to have lunch was used in another Portuguese study with a national representative sample, motivates the researchers to that procedure. The use of a convenience sample determined a higher proportion of non-academic staff as they were more available for data collection.

## 5. Conclusions

The most important determinants identified by respondents choosing the place for having meals were “meal quality”, “price”, and “location/distance”. For women, the availability of “healthy food options” was more important than for men.

Our results seem to demonstrate that gender, marital status, academic degree and main professional occupation, are related to the choice of the place for having lunch. Differences were found between gender, marital status and age ranges, in terms of factors-affecting food choice at the workplace. A higher concern with nutritional value of food was observed for younger respondents, individuals living alone, and women.

Gender and academic degree are relevant in food choice. Factors influencing individuals with a low academic degree were previous food habits, price, and quality of meals, in determining the choice of place for having lunch at restaurants or at home. On the other hand, women with a high academic degree prefer to bring meals from home as they find them healthier.

Related to determinants of food choice in general, MCA analysis reported the major differences related to academic degree and main occupation, with lower academic degree individuals being not influenced by external determinants, since their food choice was mainly influenced by previous food habits. Higher academic degree employees in general are influenced by nutritional value of food and its relationship to health and well-being, packaging, and health professional advice, the reason why strategies to promote healthy eating in these scenarios are necessarily different. If we could design a healthy eating program based on information about the nutrition value of food and health, namely through packaging, our results would show clearly that this option could be adequate for teachers and other employees with high academic degrees, but not for others that probably need personal counseling to change previous food habits.

This work also identified lack of time, work commitments, and lack of healthy options for having meals at the workplace as barriers for healthy eating. Educational level, professional occupation, and gender were the socio-economic characteristics evaluated that influenced the perception of barriers for healthy eating.

These results may contribute to a better definition of strategies to promote healthy eating in these scenarios and show that different strategies are needed for different target groups to reduce barriers once they are perceived differently by individuals.

## Figures and Tables

**Table 1:** Food consumption determinants to choose the place for having lunch per age group.

Determinants for Place Choosing	Youngers (%)	Olders (%)	*p*-Value
Price	24.1	20.4	0.006 ^1^
Meal quality	21.0	20.4	0.457
Location	15.2	18.3	0.104
Healthy food options	13.1	13.2	0.859
Lead time	11.0	9.7	0.298
Social relationships	7.4	8.9	0.354
Variety of menu	4.8	4.3	0.561
Noise	1.9	2.8	0.282
Others	1.1	1.6	0.439
Common use with students	0.4	0.5	0.765

^1^ Differences with statistical significance.

**Table 2:** Perceived barriers for healthy eating at the workplace by gender.

Barriers for Healthier Eating	Male (%)	Female (%)	*p*-Value
Do not like healthy food	3.7	6.7	0.449
Price of healthy foods	8.3	6.7	0.420
Inability to prepare meals	10.2	9.2	0.526
Lack of knowledge about nutrition/healthy eating	12.0	5.0	0.019 ^1^
Distance to food stores	5.6	1.7	0.067
Work commitments/lack of time	22.2	31.9	0.222
Lack of storage facilities and food preparation at the workplace	5.6	9.2	0.436
Lack of healthy options for breakfast, lunch and dinner	19.4	16.8	0.244
Special nutritional needs	0.9	1.7	0.707
This subject does not interest me/I never though this matter	4.6	3.4	0.466
Others	7.4	7.6	0.781

^1^ Differences with statistical significance.

**Table 3:** Perceived barriers for healthy eating at the workplace by age group.

Barriers for Healthier Eating	Youngers (%)	Olders (%)	*p*-Value
Do not like healthy food	6.1	4.2	0.288
Price of healthy foods	5.3	10.5	0.308
Inability to prepare meals	11.4	7.4	0.104
Lack of knowledge about nutrition/healthy eating	9.8	6.3	0.131
Distance to food stores	3.8	3.2	0.561
Work commitments/lack of time	28.0	26.3	0.101
Lack of storage facilities and food preparation at the workplace	7.6	7.4	0.577
Lack of healthy options for breakfast, lunch and dinner	17.4	18.9	0.575
Special nutritional needs	0.8	2.1	0.499
This subject does not interest me/I never though this matter	1.5	7.4	0.058
Others	8.3	6.3	0.279

**Table 4:** Perceived barriers for healthy eating at the workplace by professional occupation.

Barriers for Healthier Eating	Academics (%)	Non-Academics (%)	*p*-Value
Do not like healthy food	3.5	6.6	0.406
Price of healthy foods	1.2	10.9	0.004 ^1^
Inability to prepare meals	7.1	10.9	0.307
Lack of knowledge about nutrition/healthy eating	5.9	10.2	0.314
Distance to food stores	5.9	2.2	0.147
Work commitments/lack of time	32.9	24.8	0.054
Lack of storage facilities and food preparation at the workplace	5.9	6.6	0.826
Lack of healthy options for breakfast, lunch and dinner	25.9	14.6	0.012 ^1^
Special nutritional needs	1.2	1.5	0.856
This subject does not interest me/I never though this matter	5.9	2.9	0.272
Others	4.7	8.8	0.236

^1^ Differences with statistical significance.

## Data Availability

The work was a part of João Lima’s doctoral thesis.
